# Green manufacturing of amoxicillin trihydrate: a malic acid-assisted crystallization framework enhanced by Taguchi–ANN optimization

**DOI:** 10.1039/d5ra09898j

**Published:** 2026-03-17

**Authors:** M. Fatih Ergin, Hasniye Yaşa, Hülya Çelik Onar

**Affiliations:** a Engineering Faculty, Department of Chemical Engineering, Istanbul University-Cerrahpaşa 34320 Avcılar Istanbul Turkey mfergin@iuc.edu.tr; b Engineering Faculty, Department of Chemistry, Istanbul University-Cerrahpaşa 34320 Avcılar Istanbul Turkey

## Abstract

Industrial production of amoxicillin trihydrate (AMCT) often suffers from low yield, impurity inclusion, and inconsistent crystal morphology. This study introduces a scalable green crystallization strategy using malic acid as a biodegradable habit modifier, developed as part of an improved eco-friendly AMCT manufacturing framework. A hybrid optimization approach integrating Taguchi design with Artificial Neural Network (ANN) modeling was employed to capture both linear and nonlinear interactions among critical process variables. Multi-technique characterization (XRD, FTIR, DSC, BET, LC-MS) confirmed that malic acid preserves lattice integrity while substantially refining particle attributes, reducing crystallite size from 85.9 to 66.4 nm and increasing specific surface area from 5.27 to 11.07 m^2^ g^−1^. This significant increase in surface area is a key physical factor theoretically favoring improved dissolution kinetics. The ANN model exhibited excellent predictive performance (*R*^2^ > 0.99) for both purity and yield. Under optimized conditions (2.5 M malic acid, pH 5.5, 60 min, 1500 rpm), AMCT crystals were obtained with 99.21% purity and 61.82% yield. These results demonstrate a robust, data-driven framework for sustainable AMCT production, providing a high-performance alternative to conventional mineral-acid-based crystallization methods.

## Abstract 2

Malic acid-assisted crystallization combined with Taguchi–ANN optimization improves yield, purity, and crystal quality of amoxicillin trihydrate.

## Graphical abstract

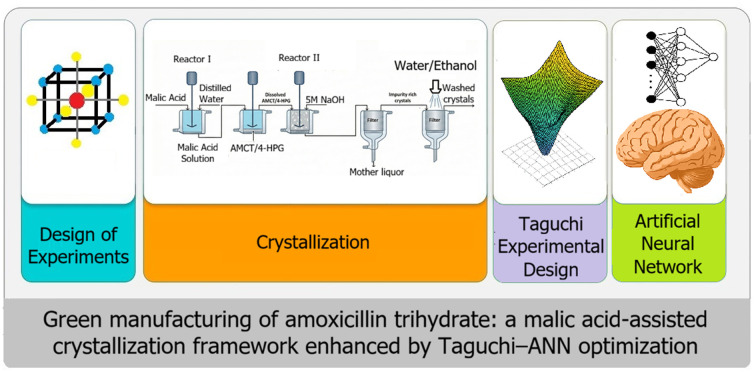

## Introduction

Amoxicillin trihydrate (AMCT) is a widely used β-lactam antibiotic essential in the treatment of Gram-positive and Gram-negative bacterial infections. The crystallization process plays a decisive role in determining the production efficiency, downstream processing, and overall pharmaceutical quality of AMCT. Key crystal attributes—such as size, morphology, and purity—directly influence the pharmacokinetics and pharmacodynamics of active pharmaceutical ingredients (APIs).^1–3^ More than 70% of APIs in the pharmaceutical industry are manufactured *via* crystallization.^4,5^ Given its global demand and classification by the World Health Organization (WHO) as a critically important antimicrobial, recent supply shortages have raised concerns about the sustainability of current production practices.^6,7^

Traditional crystallization methods often rely on toxic solvents and energy-intensive conditions, rendering them incompatible with green chemistry goals. In this context, biodegradable and non-toxic organic acids such as malic acid offer promising alternatives. Malic acid facilitates AMCT crystallization under mild conditions, improving crystal morphology and yield while minimizing impurity formation.^8–12^ Compared to conventional mineral acids like hydrochloric acid (HCl)—which is corrosive and environmentally harmful—malic acid presents a safer, biodegradable, and regulatory-compliant solution.^13–15^

Despite growing interest in alternative crystallization agents, the design of optimized crystallization protocols remains limited by conventional one-variable-at-a-time approaches or empirical methods. Taguchi experimental design provides a statistically robust, low-cost method to identify influential parameters with minimal trials.^16–19^ However, it lacks the capability to model nonlinear relationships or predict beyond fixed experimental levels. In contrast, artificial neural networks (ANNs) excel at modeling complex, nonlinear interactions but require large, diverse datasets and careful training to avoid overfitting^20–26^

In addition, degradation products such as 4-hydroxyphenylglycine (4-HPG) and 6-aminopenicillanic acid (6-APA) are known to negatively impact crystallization yield and purity, further complicating optimization efforts. Therefore, comprehensive parameter control—pH, stirring speed, crystallization time, and acid concentration—is critical. Accurate detection of these impurities is essential for process validation, as demonstrated by recent green spectroscopic methods developed by our group.^27,28^

While other organic acids such as citric acid have also been studied as crystallization modifiers, detailed engineering insights and predictive modeling remain scarce. In a prior study, our group demonstrated the empirical benefits of using malic acid for AMCT crystallization *via* factorial experimental design.^16,29,30^ However, that work lacked in-depth structural characterization, comparative analysis with other organic acids, and predictive optimization tools required for industrial scalability.

This study addresses these limitations by proposing a novel, scalable “green manufacturing framework” for AMCT production. Unlike routine optimization efforts, this work integrates a dual-patented crystallization methodology utilizing malic acid as a specific habit modifier; Patents TR 2022 017 748 A2 & TR 2023 019 116 A2 (ref. 27 and 31) with a hybrid Taguchi–ANN intelligence model. This combined approach allows for precise prediction of yield and purity within a continuous design space, minimizing experimental waste. Furthermore, we provide a comparative morphological analysis against citric acid (using BET and XRD), proving the unique industrial efficacy of the patented malic acid process. By replacing HCl with a biodegradable agent and utilizing ANN-driven optimization, this study contributes directly to the development of sustainable, reproducible, and GMP-compliant pharmaceutical manufacturing protocols.

## Materials and methods

### Materials

Amoxicillin trihydrate (AMCT) of pharmaceutical grade was purchased from North China Pharmaceutical Inc. (Hebei, China) and used without further purification. Malic acid (Merck) was selected as the primary green crystallization agent due to its natural origin and biodegradable characteristics. Citric acid (Merck) was also procured to serve as a comparative benchmark for morphological assessments. Additional reagents and solvents—including 4-hydroxyphenylglycine (4-HPG), ethanol, monopotassium phosphate (KH_2_PO_4_), and dibasic potassium phosphate (K_2_HPO_4_)—were obtained from Sigma-Aldrich (analytical grade). Sodium hydroxide (NaOH, Sigma-Aldrich) was used to prepare a 5 M stock solution for pH adjustment during crystallization. All chemicals were used as received unless otherwise stated.

## Methods

### Optimized crystallization process of amoxicillin trihydrate using malic acid

The crystallization process was executed in three main stages: (1) preparation of the malic acid solution, (2) crystallization, and (3) filtration and drying. Malic acid solutions (1.5, 2.0, and 2.5 M) were freshly prepared in an ultrasonic bath immediately prior to use to prevent light-induced degradation and stored in the dark at room temperature (∼22–25 °C).

**Fig. 1:**
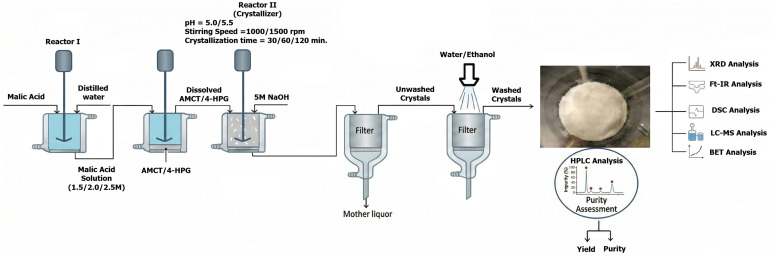
Schematic representation of the malic acid-assisted crystallization process for AMCT.

Experiments were performed in 100 mL jacketed glass reactors as illustrated in Fig. 1. For each trial, the malic acid solution was added to a mixture of AMCT (1.67 g) and 4-HPG (0.167 g). The mixture was stirred until complete dissolution (initial pH 1.7–1.9) and equilibrated for 5 minutes. Crystallization was induced by the gradual addition of 5.0 M NaOH (1 mL min^−1^) under continuous pH monitoring. The experimental design investigated four key parameters: malic acid concentration (1.5–2.5 M), pH (5.0–5.5), stirring speed (1000–1500 rpm), and time (30–120 min), building upon previous kinetic studies.^13,14,27,32^

Crucially, upon completion, the crystals were collected by vacuum filtration and washed thoroughly with 10 mL ethanol–water (1 : 1, v/v). This washing step ensures the complete removal of residual malic acid and surface impurities, confirming that malic acid functions solely as a process aid and habit modifier without being incorporated into the final crystal lattice. The final product was dried in a desiccator. Each experiment was performed in triplicate.

While citric acid was also evaluated as a green acid in selected control experiments, preliminary screening revealed that malic acid exhibited significantly superior performance in modifying crystal habit and increasing surface area. Therefore, to ensure computational efficiency and focused process development, all statistical modeling and optimization (Taguchi and ANN) efforts were concentrated exclusively on the malic acid-assisted process. AMCT recrystallized using citric acid samples (AMCT–recC) were used strictly as a structural benchmark to highlight the specific advantages of the developed malic acid framework. Comparative characterization results are provided in the SI (Fig. S1–S4).

### High-performance liquid chromatography (HPLC) analysis for purity and yield determination

Quantitative analysis of purity and yield was performed using a high-performance liquid chromatography (HPLC) system (Shimadzu 1100). Calibration curves were established using pharmaceutical-grade AMCT and 4-hydroxyphenylglycine (4-HPG) standards to ensure precise quantification of both the active ingredient and the primary degradation impurity.

Separation was achieved using a 5 µm Alltech Econosil C-18 column (250 mm × 4.6 mm). The mobile phase consisted of (A) a methanol–acetonitrile mixture (3 : 1, v/v) and (B) a 0.05 M phosphate buffer solution (pH 5.9). The buffer was prepared by dissolving 10 mL of 0.2 M K_2_HPO_4_ and 90 mL of 0.2 M KH_2_PO_4_ in 1000 mL of deionized water, followed by filtration through a 0.45 µm membrane and degassing in an ultrasonic bath prior to use.

The analysis was conducted at a flow rate of 1.0 mL min^−1^ with UV detection at 230 nm and an injection volume of 10 µL. The elution followed a binary gradient program as detailed in Table 1, with a total runtime of 15 minutes per sample.

**Table 1:** Binary gradient elution profile used for HPLC analysis

Time (min)	Mobile phase B (%) (phosphate buffer)	Mobile phase A (%) (methanol : acetonitrile)
0	0	100
2.5	10	90
4	40	60
5	20	80
7	10	90
15	0	100

Post-crystallization, the chemical purity (%) of AMCT was calculated using the area normalization method relative to the reference standard, as shown in eqn (1).1
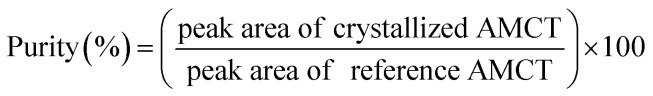


The crystallization yield (%) was determined gravimetrically using eqn (2):2
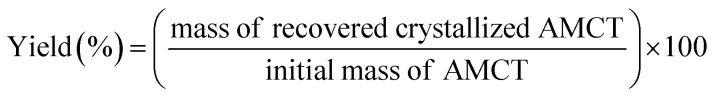


### X-ray diffraction (XRD) analysis of malic acid-assisted amoxicillin trihydrate crystals

X-ray diffraction (XRD) analysis was performed to evaluate phase purity and crystallite dimensions using a Bruker S4 Explorer spectrometer with Cu-Kα radiation (*λ* = 1.5406 Å). Scans were conducted over a 2*θ* range of 5° to 40°. To quantify the effect of the green solvent on crystal growth, the average crystallite size (*D*) was calculated based on peak broadening using the Scherrer equation:3
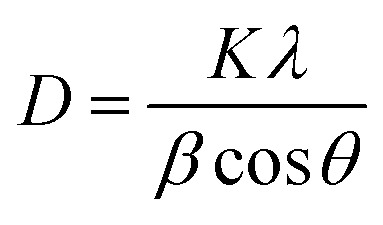
where *K* is the shape factor (typically 0.9), *λ* is the X-ray wavelength, *β* is the full width at half maximum (FWHM) of the diffraction peak, and *θ* is the Bragg angle. For benchmarking purposes, AMCT–recC was analyzed under identical conditions to compare the morphological impact of different organic acids. Comparative diffractograms are provided in the SI (Fig. S1).

### Fourier-transform infrared spectroscopy (FTIR) analysis

Fourier-transform infrared (FTIR) spectroscopy was employed to verify molecular integrity and investigate potential chemical interactions—such as hydrogen bonding or salt formation—between AMCT and the organic acids during crystallization. Spectra were acquired using a JASCO 6300 spectrophotometer equipped with an Attenuated Total Reflectance (ATR) accessory. Data were collected in the wavenumber range of 4000–400 cm^−1^ with a resolution of 4 cm^−1^, averaging 32 scans per sample.

The following samples were analyzed to track structural changes:

• Pure AMCT (reference),

• Pure malic acid and pure citric acid,

• AMCT recrystallized from malic acid (AMCT–recM),

• AMCT recrystallized from citric acid (AMCT–recC).

Spectral analysis focused on critical functional groups, specifically the fingerprint region (1500–600 cm^−1^), the carbonyl stretching region (∼1730–1650 cm^−1^), and the hydroxyl/amine regions (∼3400–3200 cm^−1^). The absence of peak shifts or new absorption bands in these regions was used as the primary criterion to confirm that no co-crystals were formed and that the green solvent served strictly as a process aid. Comparative spectra are provided in the SI (Fig. S2).

### Differential scanning calorimetry (DSC) analysis

Differential scanning calorimetry (DSC) was utilized to evaluate thermal stability and verify phase purity by comparing the thermal profiles of the raw materials and the crystallized products. Analyses were performed using an Exstar 6200 DSC system (SII Nanotechnology, Japan).

Samples (approximately 2–3 mg) were encapsulated in sealed aluminum pans and subjected to a heating ramp from 30 °C to 200 °C at a rate of 10 °C min^−1^ under a continuous nitrogen purge to prevent oxidation. The analysis included:

• Pure AMCT (reference),

• Pure malic acid and pure citric acid,

• AMCT–recM,

• AMCT–recC.

Thermograms were analyzed to detect endothermic transitions associated with dehydration and melting. The primary criterion for process validation was the absence of new thermal events (such as eutectic melting) or significant shifts in the AMCT melting peak, which would otherwise indicate co-crystal formation or acid inclusion. Comparative thermograms confirming the structural integrity of the products are provided in the SI (Fig. S3).

### Liquid chromatography-mass spectrometry (LC-MS) analysis

Liquid chromatography-mass spectrometry (LC-MS) was performed to verify the molecular composition of the recrystallized samples and to conclusively rule out the formation of malic acid co-crystals or adducts. The analysis was conducted using a Shimadzu LC-MS system equipped with an electrospray ionization (ESI) source operating in negative ion mode. The mass spectrometer was calibrated to scan the *m*/*z* range of 100–1000. Recrystallized AMCT samples were dissolved in methanol–water (1 : 1, v/v), filtered through 0.22 µm PTFE syringe filters, and directly injected. The mass spectrum of AMCT–recM exhibited a characteristic deprotonated molecular ion [M–H]^−^ at *m*/*z* 364, corresponding to pure amoxicillin1. Crucially, no additional signals attributable to malic acid adducts, co-crystal species, or other high-molecular-weight impurities were detected. These results confirm that malic acid does not participate in any molecular association or structural incorporation, validating its role solely as a process solvent. Comparative analysis of AMCT–recC yielded identical spectral results, as shown in the SI (Fig. S4).

### Brunauer–Emmett–Teller (BET) surface area analysis

To quantify the morphological impact of the green crystallization agents on particle surface characteristics, specific surface area measurements were conducted using the Brunauer–Emmett–Teller (BET) method based on nitrogen gas adsorption–desorption isotherms. Analyses were performed using a Quantachrome NOVA instrument utilizing NovaWin software (v11.03).

Samples (approximately 0.44 g) were prepared for analysis under strict conditions to maintain crystal integrity. Crucially, to prevent the dehydration of the amoxicillin trihydrate structure, no thermal degassing was applied. Instead, samples were subjected to a vacuum during the automated preparation stage to remove weakly adsorbed surface species without disturbing the lattice water. Nitrogen was used as the adsorbate gas at a bath temperature of 77 K.

The BET surface area was calculated from the linear region of the adsorption isotherm within the relative pressure (*P*/*P*_0_) range of 0.05 to 0.35. The analysis compared three distinct samples to assess the engineering efficacy of the additives:

• Pure AMCT (reference),

• Pure malic acid and pure citric acid,

• AMCT–recM,

• AMCT–recC.

### Taguchi experimental design: optimization of crystallization parameters using malic acid

To systematically optimize the green crystallization process, a Taguchi experimental design was employed. Based on initial kinetic screening, four critical process variables were identified: malic acid concentration, stirring speed, pH, and crystallization time.

A Taguchi L18 orthogonal array was constructed to efficiently screen the design space, minimizing experimental workload while maximizing statistical reliability. The complete experimental matrix is provided in Table S1 (SI). The factors and their corresponding levels (Table 2) were selected to maximize the removal of the primary impurity (4-HPG) while enhancing crystal yield.

**Table 2:** Process variables and levels used in the Taguchi experimental design

Process parameters	Level 1	Level 2	Level 3
Malic acid concentration	1.5 M	2.0 M	2.5 M
Stirring speed	500 rpm	1000 rpm	1500 rpm
pH	5.0	5.5	—
Crystallization time	30 min	60 min	120 min

To evaluate the optimization objective, the experimental results were transformed into a Signal-to-Noise (S/N) ratio. Since the goal of this study is to maximize both production yield and product purity, the “larger-is-better” criterion was adopted. The S/N ratio is calculated using eqn (4):4
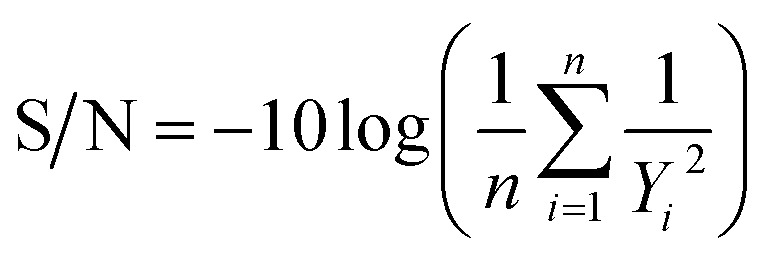


where *Y*_*i*_ represents the measured response (yield or purity) for the *i*-th experiment, and *n* is the number of repetitions. The statistical significance and percentage contribution of each parameter were subsequently determined using Analysis of Variance (ANOVA).

### Artificial neural network (ANN) model for predicting and optimizing crystallization parameters

To transcend the linear limitations of traditional statistical methods and capture complex non-linear interactions between process variables, an Artificial Neural Network (ANN) model was developed. The model was designed to predict two critical quality attributes (CQAs)—yield and purity—based on four input process parameters: stirring speed, pH, crystallization time, and malic acid concentration.

**Fig. 2:**
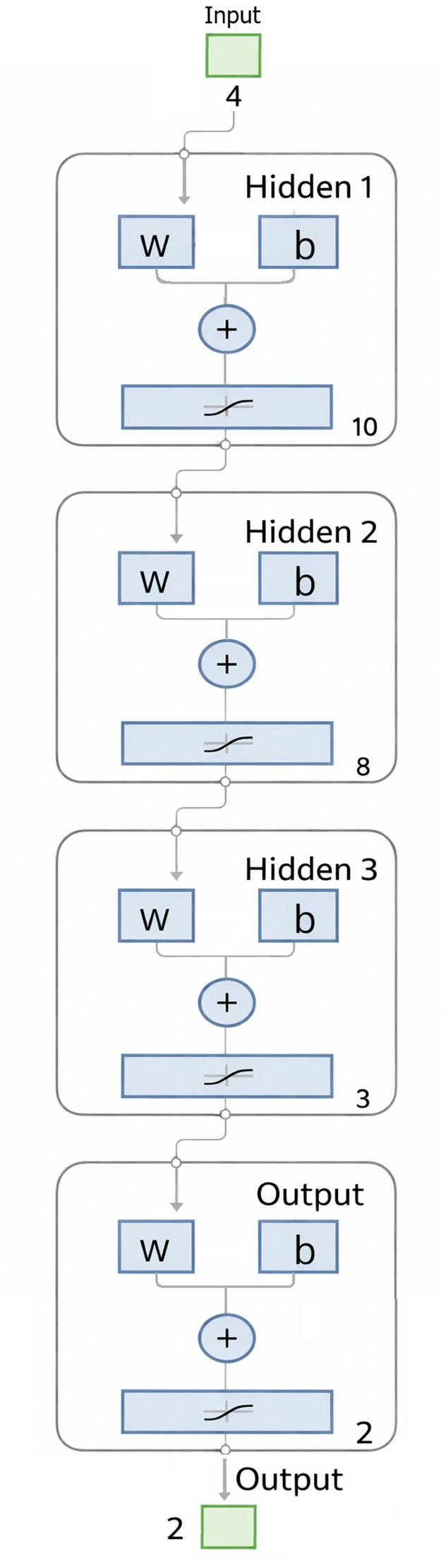
Architecture of the multilayer ANN used for predicting AMCT yield and purity.

The network topology, implemented in MATLAB R2024a, consisted of a multilayer feed-forward architecture with three hidden layers containing 10, 8, and 3 neurons, respectively, as illustrated in Fig. 2. This specific configuration was selected after a heuristic optimization process to balance computational efficiency with the ability to model complex topologies without overfitting.

To ensure robust generalization, the experimental dataset (derived from the Taguchi L18 array and extended with 36 additional independent batch experiments to form 54 data pairs, see Table S2) was randomly partitioned into training (70%), validation (15%), and testing (15%) subsets. The Levenberg–Marquardt backpropagation algorithm was employed for training, targeting a Mean Squared Error (MSE) minimization.

Crucially, to mitigate the risk of overfitting inherent in smaller datasets, strict regularization techniques were applied:

• Early stopping: training was automatically halted when validation error ceased to decrease.

• Cross-validation: the high coefficient of determination (*R*^2^ > 0.99) achieved across all subsets confirms that the model learned the underlying process physics rather than memorizing noise.

It is important to note that the ANN model is calibrated strictly within the experimental design space defined by the Taguchi method. While highly accurate for interpolation, the model is not intended for extrapolation beyond the tested parameter ranges.

### ANN-enhanced Taguchi strategy for efficient crystallization of amoxicillin trihydrate

To bridge the gap between discrete statistical screening and continuous process modeling, a hybrid optimization strategy was implemented. The experimental framework was anchored by a Taguchi L18 (2^1^ × 3^3^) orthogonal array, designed to screen four independent variables: pH (5.0, 5.5), stirring speed (500, 1000, 1500 rpm), crystallization time (30, 60, 120 min), and malic acid concentration (1.5%, 2.0%, 2.5% w/v).

While the Taguchi design efficiently identified significant factors and Signal-to-Noise (S/N) ratios, it is inherently limited to linear approximations at discrete levels. To overcome this, the dataset was systematically enriched. The 18 Taguchi runs served as the “skeleton” of the design space, which was then supplemented with 36 additional independent batch experiments targeting intermediate combinations. These supplementary data points were experimentally obtained within the same design space to provide high-resolution data for robust ANN training, ensuring that the model learns from physical process variations rather than synthetic trends. This comprehensive experimental approach allowed the ANN model to perform precise non-linear mapping across the entire experimental domain, strictly avoiding unreliable extrapolation.

The resulting dataset (54 experimental points) was used to train a multilayer feed-forward backpropagation network. Unlike basic single-layer models, a topology with three hidden layers was employed to capture complex non-linear crystallization kinetics. Robustness was ensured *via* Bayesian regularization and Y-randomization tests, preventing the overfitting often associated with limited datasets.

This synergy provides a dual engineering advantage: the Taguchi method minimizes experimental waste by identifying the design boundaries, while the ANN transforms this high-density experimental data into a predictive “digital twin” of the process, enabling precise control over yield and purity.

## Results and discussion

### Structural and morphological characterization of AMCT crystals

To validate the proposed green manufacturing framework, a comprehensive multi-technique characterization was performed to assess phase purity, molecular integrity, and morphological evolution.

### Crystallinity and lattice integrity (XRD)

**Fig. 3:**
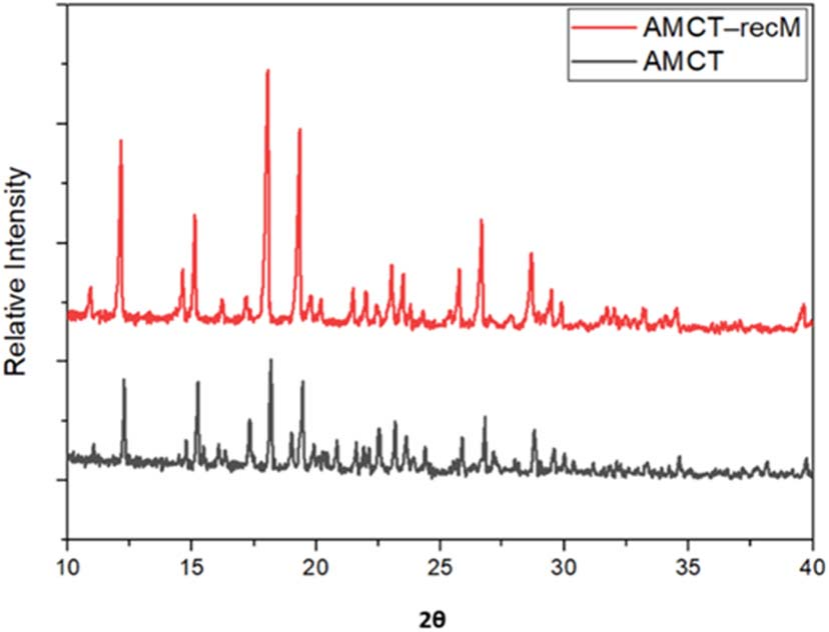
XRD patterns of pure AMCT and AMCT–recM. Notable peak shifts and enhanced crystallinity demonstrate the structural influence of malic acid.

X-ray diffraction (XRD) analysis revealed significant structural refinement in AMCT–recM compared to pure AMCT (Fig. 3). While the characteristic diffraction peaks of AMCT were preserved—confirming that the crystal lattice structure remained chemically intact—notable peak sharpening and intensity increments were observed, particularly at 2*θ* = 12.13° and 18.03°. These changes indicate enhanced crystallinity and lattice ordering. Detailed crystallographic parameters, including peak positions, *d*-spacing values, and relative intensities for all samples, are provided in Table S3 (SI).

Crucially, no new peaks attributable to malic acid co-crystals or salt forms were detected, supporting the premise that malic acid acts strictly as a habit modifier rather than a lattice component. The crystallite size, calculated *via* the Scherrer equation, decreased from 85.9 nm (pure AMCT) to 66.4 nm (AMCT–recM). This reduction confirms that malic acid effectively suppresses excessive crystal growth while promoting nucleation, a key mechanism for improving powder processability. These findings are consistent with particle size control strategies described in literature, where organic additives act as habit modifiers to tailor crystal size distributions.^8,11,33^

Additionally, a comparative XRD evaluation between AMCT–recM and AMCT–recC is provided in the SI (Fig. S1). This comparison further illustrates that malic acid uniquely induces significant structural enhancements superior to those of citric acid, validating its selection as the primary agent for this optimization framework.

### FTIR spectroscopy: functional group and interaction assessment

**Fig. 4:**
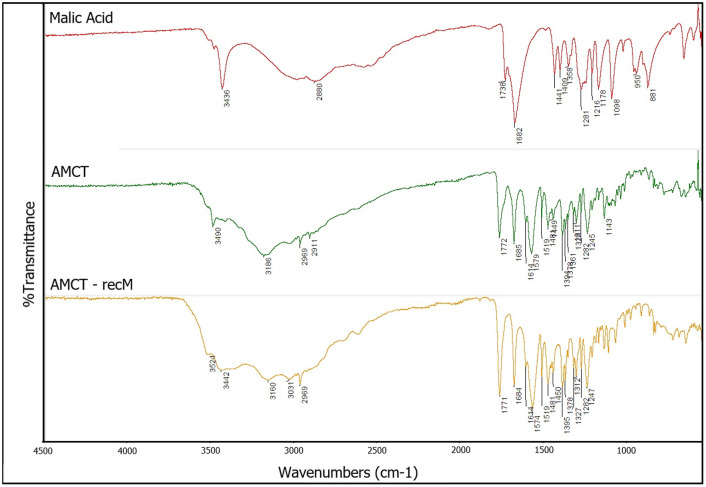
FTIR spectra of pure AMCT, pure malic acid, and AMCT–recM (recrystallized from malic acid). The preserved carbonyl (∼1730–1650 cm^−1^) and hydroxyl/N–H stretching regions (∼3400–3200 cm^−1^) confirm the absence of interaction or co-crystal formation.

Fourier-transform infrared (FTIR) spectroscopy was employed to comprehensively assess potential molecular interactions and to verify that the green solvent did not compromise the chemical integrity of the active pharmaceutical ingredient. Spectra were recorded for pure AMCT, pure malic acid, and the recrystallized product AMCT–recM (Fig. 4).

As clearly demonstrated in Fig. 4, the characteristic FTIR absorption bands of AMCT remained intact and unaffected in the AMCT–recM sample. Notably, the critical carbonyl stretching region (∼1730–1650 cm^−1^), which corresponds to the lactam structure crucial for antibiotic activity, as well as the broad N–H and O–H stretching bands (∼3400–3200 cm^−1^), exhibited no shifts or new absorption features. Recent studies emphasize the reliability of FTIR fingerprinting in detecting subtle structural modifications or degradation in β-lactam antibiotics, confirming that spectral rigidity is a definitive indicator of molecular stability.^34^ This absence of spectral changes provides robust evidence that no significant hydrogen bonding, salt formation, or co-crystal structures were formed during recrystallization.

Furthermore, the fingerprint region (1500–600 cm^−1^), which is particularly sensitive to molecular conformational changes, confirmed the structural stability of AMCT. These results collectively validate the hypothesis that malic acid acts purely as an environmentally friendly process aid without chemically incorporating into the AMCT crystal lattice. Unlike co-crystal systems where distinct peak shifts are observed due to non-covalent interactions such as hydrogen bonding networks,^5,26,35^ the absence of such shifts in AMCT–recM confirms the preservation of the original active pharmaceutical ingredient structure. Comparative FTIR spectra for AMCT–recC are provided in SI (Fig. S2), further supporting the inert role of the employed organic acids.

### DSC analysis: thermal behavior and hydration loss

**Fig. 5:**
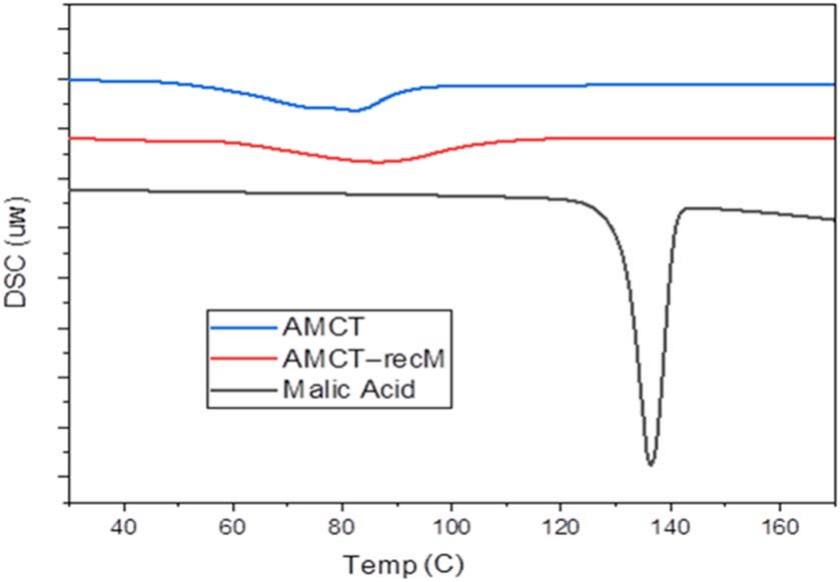
DSC thermograms of pure AMCT, AMCT–recM, and pure malic acid. The dehydration and melting events of AMCT remain consistent after recrystallization, while the absence of malic acid's thermal peak confirms no chemical incorporation.

Differential scanning calorimetry (DSC) analysis was performed to investigate the thermal characteristics and hydration stability of pure AMCT and AMCT–recM. Thermograms of pure AMCT, AMCT–recM, and pure malic acid are shown comparatively in Fig. 5.

The pure AMCT sample exhibited a characteristic broad endothermic peak between 50–100 °C, corresponding to the dehydration of the trihydrate form, followed by a distinct sharp melting peak at approximately 163 °C. These thermal transitions are consistent with the characteristic decomposition kinetics of amoxicillin trihydrate reported in recent stability studies.^34^

These thermal transitions remained unchanged in the thermogram of AMCT–recM, strongly suggesting that recrystallization with malic acid did not alter the crystalline integrity or hydration properties of AMCT. Crucially, in the DSC thermogram of pure malic acid, a clear melting event was observed at approximately 133–137 °C. This characteristic peak was notably absent in the AMCT–recM thermogram, providing definitive evidence that malic acid molecules were effectively removed during the washing step (section Optimized crystallization process of amoxicillin trihydrate using malic acid) and were not incorporated into the crystal lattice.

Furthermore, the absence of additional thermal events or peak shifts in the AMCT–recM thermogram confirms that no co-crystal formation, polymorphic transformation, or significant molecular interactions occurred. Literature on binary phase diagrams confirms that the formation of a pharmaceutical cocrystal is typically characterized by a distinct melting endotherm differing from the individual components; the absence of such a new peak in the DSC thermogram is a recognized indicator of phase purity.^36^ These thermal analysis results align closely with the FTIR spectroscopy and LC-MS findings, collectively reinforcing the validation of malic acid as a transient process aid rather than a structural component. Comparative DSC thermograms for AMCT–recC are provided in the SI (Fig. S3), further confirming the inert nature of organic acids employed in this study.

### LC-MS analysis: molecular composition and purity verification

**Fig. 6:**
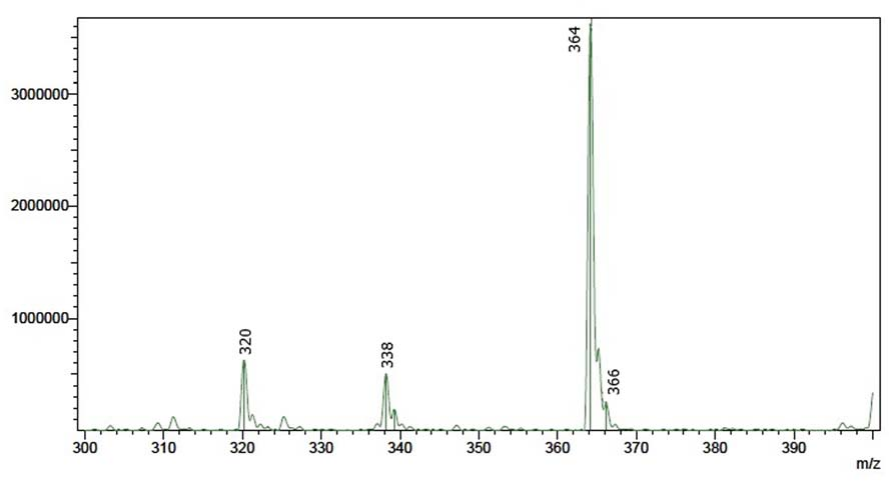
LC-MS chromatogram of AMCT–recM, showing a single dominant peak at *m*/*z* 364 corresponding to [M–H]^−^ of amoxicillin trihydrate. The absence of secondary peaks confirms the structural integrity and purity of the recrystallized product.

Liquid chromatography-mass spectrometry (LC-MS) analysis was conducted to evaluate the molecular integrity and purity of recrystallized AMCT samples, and specifically to determine if any chemical interactions occurred between AMCT and malic acid during recrystallization. As clearly shown in Fig. 6, the LC-MS chromatogram of the AMCT–recM sample exhibited a single dominant peak at *m*/*z* 364, accurately corresponding to the deprotonated molecular ion [M–H]^−^ of amoxicillin trihydrate. This spectral signature is consistent with established fragmentation patterns for amoxicillin, where the *m*/*z* 364 ion serves as the primary diagnostic peak for the intact molecule in negative electrospray ionization mode.^37^

The absence of additional mass signals across the entire scanned *m*/*z* range indicates that no chemical adducts, co-crystal entities, impurities, or degradation products formed during recrystallization. Significantly, no peaks related to malic acid or its potential fragments were detected. This strongly verifies that malic acid was not chemically incorporated into the AMCT crystal lattice or structurally bound to AMCT molecules.

These LC-MS findings align precisely with the results obtained from the FTIR and DSC analyses, collectively reinforcing the conclusion that malic acid functioned purely as an eco-friendly solvent facilitating crystallization, without any chemical modification or structural interference with AMCT. Further comparative LC-MS analysis performed on AMCT–recC similarly showed no detectable acid-related peaks or interaction products, emphasizing the inert and structurally neutral role of the employed organic acids. Detailed LC-MS chromatograms for AMCT–recC are provided in the SI (Fig. S4).

### BET surface area analysis: morphological implications

Brunauer–Emmett–Teller (BET) surface area analysis was utilized to quantify the engineering efficacy of the green crystallization agents. As summarized in Table 3, pure AMCT exhibited a baseline surface area of 5.27 m^2^ g^−1^.

**Table 3:** BET surface area values of pure AMCT, AMCT–recM, and AMCT–recC samples

Sample	Surface area (m^2^ g^−1^)
Pure AMCT	5.27
AMCT–recM	11.07
AMCT–recC	5.76

Ideally, a crystallization modifier should significantly increase surface area to enhance dissolution kinetics without compromising purity. In this context, malic acid demonstrated superior performance, achieving a specific surface area of 11.07 m^2^ g^−1^ (*a* >110% increase over the reference). In sharp contrast, citric acid—used as a green benchmark—yielded only a marginal increase to 5.76 m^2^ g^−1^. This comparative data justifies the selection of malic acid as the primary agent for this optimization framework, as it offers a significantly more favorable surface-to-volume ratio.

This substantial enhancement in surface area correlates directly with the crystallite size reduction observed in XRD analysis (section Crystallinity and lattice integrity (XRD)), confirming that malic acid effectively promotes nucleation while restricting excessive crystal growth. From a pharmaceutical engineering perspective, the generation of such high-surface-area crystals is critical for improving the bioavailability of poorly water-soluble drugs like AMCT.

### Pharmaceutical implications of crystallite modifications and purity enhancement

The ability to engineer particle morphology through green solvent selection has direct implications for downstream pharmaceutical processing. The significant reduction in crystallite size and the >100% increase in specific surface area (confirmed by BET analysis) suggest that the malic acid-assisted process can enhance the dissolution kinetics of AMCT, a critical factor for the bioavailability of poorly water-soluble antibiotics.

From a process engineering perspective, the production of finer, high-purity crystals (99.21%) under optimized conditions improves downstream unit operations such as filtration and drying efficiency. Furthermore, the experimentally validated yield of 61.82% demonstrates that this green route is commercially viable, balancing mass recovery with high product quality.

Crucially, replacing corrosive mineral acids (*e.g.*, HCl) with biodegradable malic acid aligns the manufacturing process with Green Chemistry principles and Environmental, Health, and Safety (EHS) regulations. This substitution minimizes the generation of hazardous acidic waste, reducing treatment costs and operator risk.

In summary, the proposed framework—underpinned by dual-patented technology^27,31^ and AI-driven predictive modeling—offers a robust, scalable, and regulatory-compliant alternative to traditional AMCT crystallization protocols. It bridges the gap between laboratory-scale crystal engineering and smart, sustainable industrial manufacturing.

### Taguchi-based optimization of AMCT crystallization: yield and purity

#### Signal-to-noise (S/N) ratio analysis

The Taguchi method was applied to maximize both the yield and purity of AMCT crystallization, focusing on four critical process parameters: pH, stirring speed, crystallization time, and malic acid concentration. The “Larger-is-better” criterion was adopted for the Signal-to-Noise (S/N) analysis to identify robust operating conditions.

**Fig. 7:**
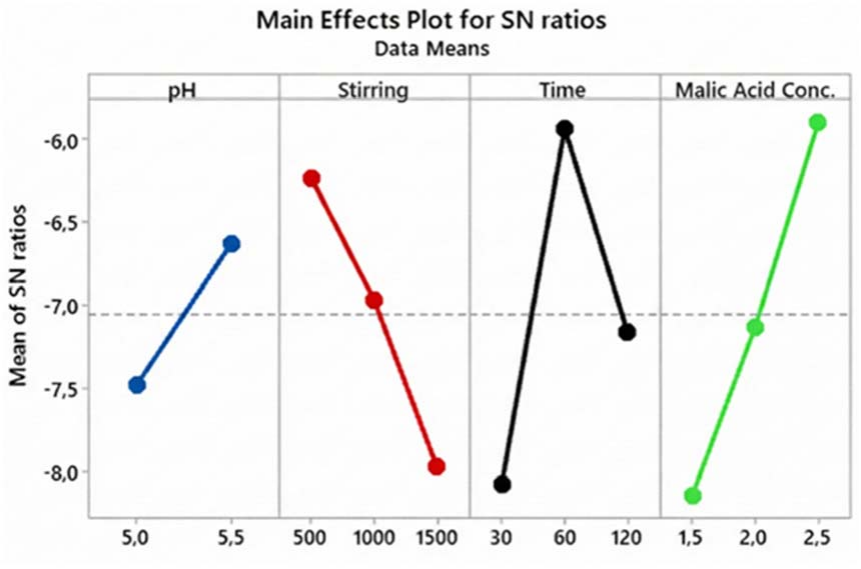
Main effects plot for S/N ratios for amoxicillin yield: influence of critical parameters on optimal conditions.

For yield optimization, the main effects plot (Fig. 7) identified the optimal settings as: pH = 5.5, stirring speed = 500 rpm, crystallization time = 60 minutes, and malic acid concentration = 2.5 M. Among these, malic acid concentration exhibited the most significant impact (Δ*a* = 2.245), confirming its critical role in driving supersaturation kinetics.

**Fig. 8:**
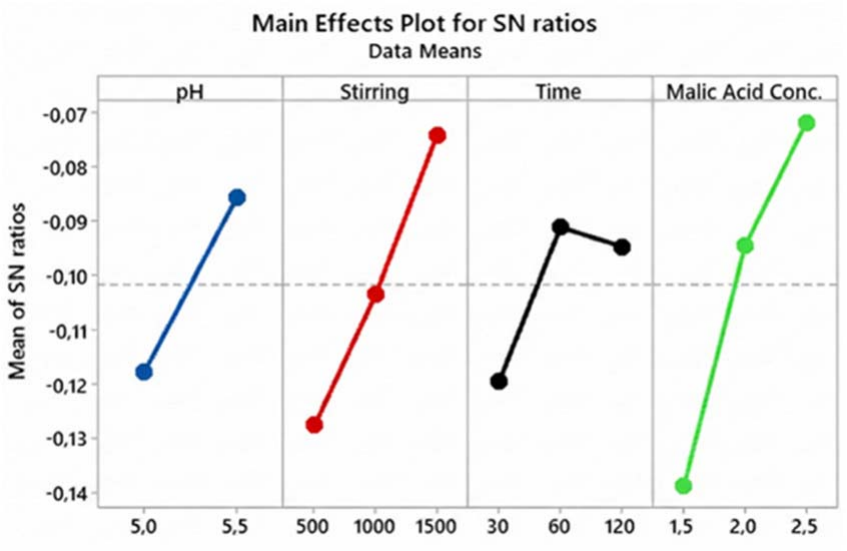
Main effects plot for S/N ratios for purity: influence of factors and optimal conditions.

For purity optimization, the optimal conditions differed slightly, favoring higher shear rates: pH = 5.5, stirring speed = 1500 rpm, crystallization time = 60 minutes, and malic acid concentration = 2.5 M. Similar to yield, malic acid concentration was the dominant factor Δ = 0.067, Fig. 8, followed by stirring speed. This suggests that a sufficiently acidic environment combined with efficient mixing is crucial for preventing impurity inclusion.

### Statistical significance (ANOVA) and model validation

Analysis of Variance (ANOVA) confirmed the statistical significance of the selected parameters. For yield, malic acid concentration (34.05%) and crystallization time (30.90%) were the dominant contributors (Table 4). For purity, malic acid concentration (39.67%) and stirring speed (24.60%) had the greatest impact (Table 5).

**Table 4:** Statistical significance of factors on Amct yield: ANOVA results

Source	DF	Seq SS	Contribution	Adj SS	Adj MS	*F*-value	*P*-value
pH	1	0.007	6.24%	0.007	0.007	8.27	0.017
Stirring	2	0.025	21.26%	0.025	0.012	14.08	0.001
Time	2	0.036	30.90%	0.036	0.018	20.47	0.000
Malic acid conc.	2	0.040	34.05%	0.040	0.020	22.55	0.000
Error	10	0.009	7.55%	0.009	0.001		
Total	17	0.117	100.00%				

**Table 5:** Statistical significance of factors on Amct purity: ANOVA results

Source	DF	Seq SS	Contribution	Adj SS	Adj MS	*F*-value	*P*-value
pH	1	0.000	13.24%	0.000	0.000	9.17	0.013
Stirring	2	0.000	24.60%	0.000	0.000	8.52	0.007
Time	2	0.000	8.05%	0.000	0.000	2.79	0.109
Malic acid conc.	2	0.000	39.67%	0.000	0.000	13.74	0.001
Error	10	0.000	14.44%	0.000	0.000		
Total	17	0.000	100.00%				

**Fig. 9:**
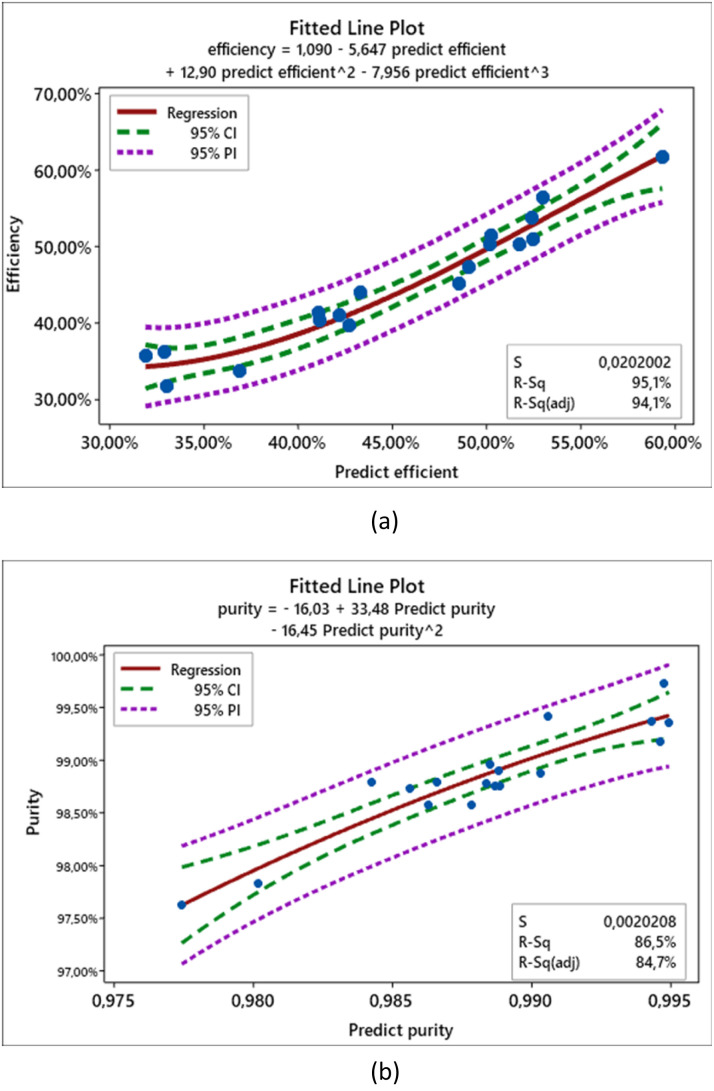
Prediction accuracy of the model. (a) Yield, (b) purity.

Regression models demonstrated strong predictive capability, with *R*^2^ values of 95.1% for yield and 86.5% for purity (Fig. 9). The models' predictions fell well within the 95% confidence and prediction intervals, reinforcing the robustness of the statistical approach.

### Interaction effects and industrial implications

**Fig. 10:**
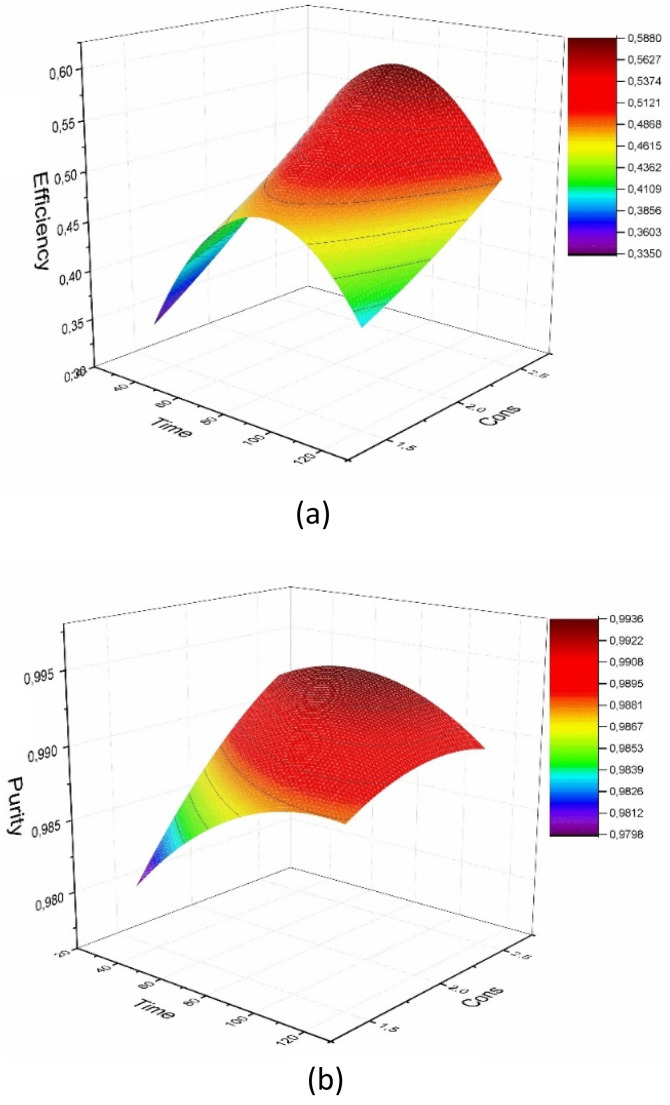
Contour plots for yield and purity: effects of concentration and time. (a) Yield, (b) purity.

Contour plots (Fig. 10) revealed significant interactions between process variables. Specifically, increasing both malic acid concentration and crystallization time led to substantial improvements in yield. However, excessively high stirring speeds (>1500 rpm) were found to disrupt crystal growth, negatively impacting yield despite favoring purity. This observation aligns with the mixing-precipitation theories suggested by ref. 17 and 16, which indicate that while intense mixing enhances mass transfer, excessive shear forces can induce secondary nucleation or crystal breakage, thereby reducing the mean crystal size and recoverable yield.

Under the experimentally validated optimal conditions (2.5 M malic acid, pH 5.5, 60 min), the process achieved a maximum yield of 61.82% and a purity of 99.21%. Because industrial AMCT manufacturing places regulatory emphasis on pharmaceutical-grade purity rather than maximum mass recovery (USP, FDA), the optimization strategy ultimately prioritized purity as the critical quality attribute. Therefore, the ANN-refined optimum conditions—favoring high shear mixing at 1500 rpm—were selected, as they consistently produced the highest purity within the validated design space. It is important to note that the yield of 61.82% represents the maximum achievable mass recovery for this specific green synthesis route, balancing high purity with acceptable industrial efficiency. These results validate the scalability of the patented malic acid–based crystallization method,^27,31^ providing a robust framework for sustainable pharmaceutical manufacturing.

### General discussion: process robustness and industrial scalability

The integrated application of the Taguchi method and ANOVA provided a systematic validation of the process parameters. Consistent with the kinetic theory of crystallization, malic acid concentration emerged as the critical process parameter (CPP), exerting the most significant influence on both yield (34.05% contribution) and purity (39.67% contribution). This statistical evidence confirms that controlling the supersaturation driving force *via* acid concentration is key to stabilizing the process.

From an industrial perspective, these findings offer a scalable and cost-effective crystallization strategy. By replacing conventional mineral acids (such as HCl) with biodegradable and pharmaceutically safe malic acid, the process aligns with Green Chemistry principles while simultaneously improving dissolution performance and process reproducibility. This substitution directly contributes to the reduction of the *E*-factor and improves the sustainability profile of the manufacturing process, as advocated in the foundational metrics of green chemistry.^15^ The elimination of corrosive reagents significantly reduces the environmental footprint and equipment maintenance costs associated with industrial-scale manufacturing.

These results highlight malic acid's dual role: functioning as a structurally neutral dissolution medium and as a functionally beneficial habit modifier. This unique capability, now validated by robust statistical modeling, positions the patented malic acid process^27,31^ as a viable, high-performance alternative for sustainable pharmaceutical manufacturing.

### Artificial neural network (ANN) analysis for yield and purity

To overcome the discrete limitations of the Taguchi method and enable continuous process monitoring, a feed-forward Artificial Neural Network (ANN) model was developed. The model was designed to predict two Critical Quality Attributes (CQAs)—yield and purity—based on four input process parameters: stirring speed, pH, crystallization time, and malic acid concentration.

### Model performance and robustness

The ANN model demonstrated exceptional predictive accuracy across all data partitions. As detailed in Table 6, the correlation coefficients (*R*) were 0.9991 for training, 0.9910 for validation, and 0.9984 for testing. The extremely low Mean Squared Error (MSE < 0.002) confirms that the model effectively captured the non-linear topology of the crystallization design space without overfitting.

**Table 6:** Performance metrics of the ANN model for training, validation, and testing datasets

Data set	MSE	*R*
Training	0.0002	0.9991
Validation	0.0014	0.9910
Test	0.0003	0.9984

### Training optimization and overfitting prevention

**Fig. 11:**
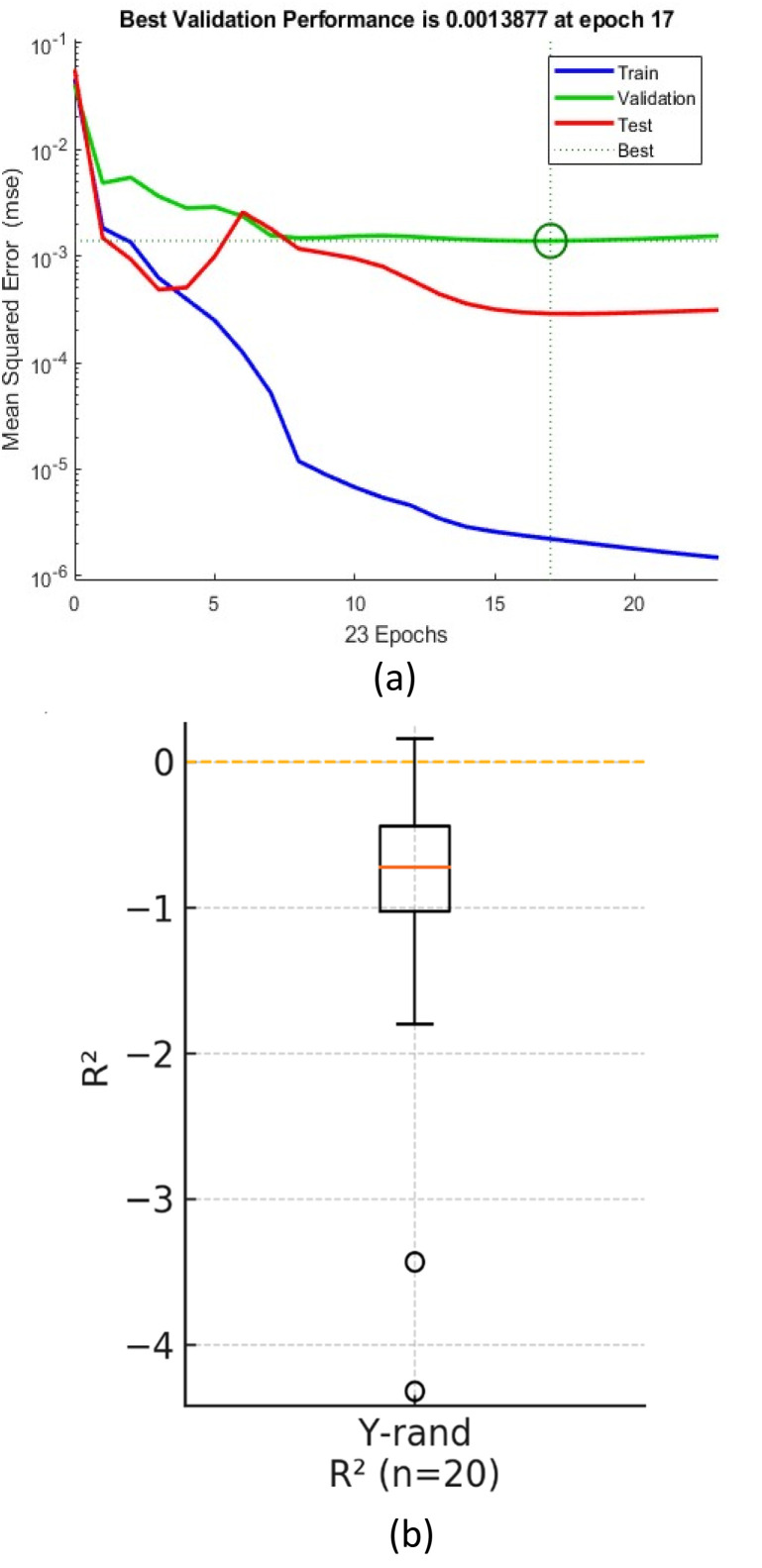
Regression plots for training, validation, and test sets: comparison between predicted and actual values for yield and purity. (a) Learning curves, (b) *R*^2^ distribution from 20 Y-randomisation trials.

To ensure the reliability of the model on unseen data, strict regularization techniques were applied. The training process reached its minimum validation loss at epoch 17 (val-MSE = 1.39 × 10^−3^), where the early-stopping algorithm automatically halted the process to preserve the optimal weights (Fig. 11a). The parallel decrease in training, validation, and test error curves indicates a well-generalized model with no signs of divergence.

Furthermore, the model's robustness was validated *via* 10-fold cross-validation (*R* = 0.988 ± 0.005) and Y-randomization tests (Fig. 11b). The randomization trials centered around zero correlation (*R*^2^ ∼0.11), confirming that the high predictive accuracy is due to genuine causal relationships between the process parameters and the outputs, not chance correlations.

### Training optimization and model robustness

**Fig. 12:**
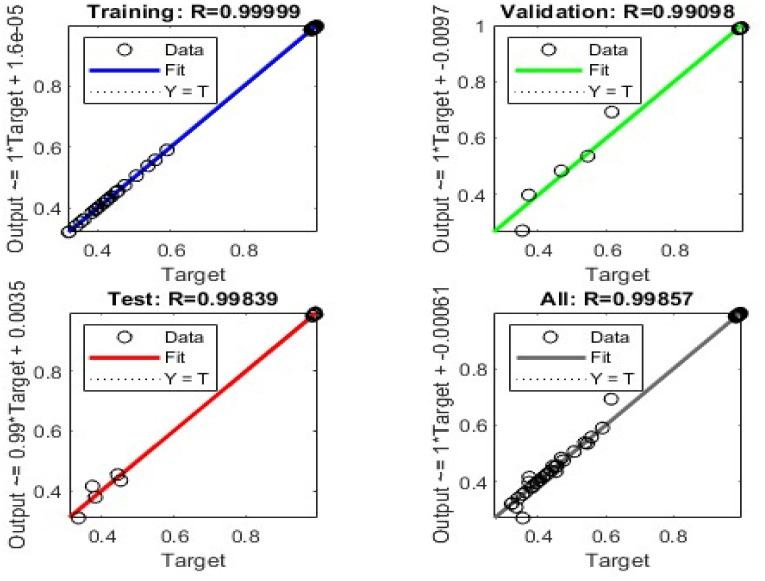
Learning curves of the ANN: train, hold-out validation, and test losses, together with 10-fold cross-validated mean ± 1 SD. The dotted line marks the early-stopping epoch 17. Inset: *R*^2^ distribution from 20 Y-randomisation runs.

The regression plots (Fig. 12) demonstrate a near-perfect linear agreement between the predicted and experimental values for both yield and purity. Unlike traditional polynomial regression, this ANN framework successfully models the complex, non-linear interactions inherent in precipitation kinetics. Recent studies emphasize the superiority of machine learning approaches over standard empirical models in capturing the multidimensional design space of pharmaceutical crystallization,^22,25^ a conclusion strongly supported by the high predictive accuracy (*R*^2^ > 0.99) achieved in this work.

From an industrial perspective, this “digital twin” capability offers significant advantages:

• Real-time optimization: it allows operators to predict batch outcomes before physical execution.

• Waste reduction: by minimizing trial-and-error experimentation, it aligns with Green Chemistry goals.

### Comparative evaluation and industrial relevance

#### Taguchi *vs.* ANN: synergistic optimization

While the Taguchi method (L18) efficiently identified the critical process parameters (pH and malic acid concentration) and their optimal discrete levels, it is inherently limited to linear approximations. The integration of ANN overcame this limitation by mapping the continuous design space. The ANN model functioned as a “digital twin,” enabling high-resolution prediction of yield and purity at intermediate conditions not tested experimentally. This synergy provides a robust framework: Taguchi ensures experimental economy, while ANN provides predictive precision.

### Comparison with literature and green manufacturing potential

The results of this study align with and extend recent findings in green crystallization. The ability of malic acid to modify crystal habit and solubility has been reported for other systems,^38,39^ but its specific application to amoxicillin morphology control is a novel contribution of this work. For instance, while previous studies on trimethoprim^40^ and calcium carbonate^41^ highlighted the role of carboxylic acids in nucleation, our study quantifies this effect for AMCT, demonstrating *a* >100% increase in surface area (section BET surface area analysis: morphological implications).

Furthermore, the use of hybrid AI models for pharmaceutical process control is gaining traction.^42,43^ Our work contributes to this trend by demonstrating that a small, well-designed dataset (Taguchi-based) is sufficient to train a high-accuracy ANN (*R*^2^ > 0.99) when proper regularization techniques are applied.

From an industrial perspective, the proposed framework offers a scalable, cost-effective, and regulatory-compliant manufacturing route. By optimizing stirring speeds and reducing acid concentrations *via* AI prediction, the process minimizes energy consumption and raw material waste, aligning with the principles of sustainable industrial crystallization.^44^

## Conclusions

This study established a novel, scalable Green Manufacturing Framework for Amoxicillin trihydrate (AMCT) production, integrating a dual-patented crystallization methodology (TR 2022 017 748 A2; TR 2023 019 116 A2) with hybrid artificial intelligence modeling. By replacing hazardous mineral acids with biodegradable malic acid, we successfully addressed key industrial challenges related to yield, purity, and environmental compliance.

The integration of Taguchi design with Artificial Neural Networks (ANN) allowed for high-precision process optimization. The ANN model demonstrated superior predictive capability (*R*^2^ > 0.99), effectively mapping the nonlinear relationships between process variables and critical quality attributes. Under the experimentally validated optimal conditions (2.5 M malic acid, pH 5.5, 60 min, 1500 rpm), the process achieved a yield of 61.82% and a purity of 99.21%.

Furthermore, advanced morphological characterization (XRD, BET, SEM) revealed that malic acid acts as a highly effective habit modifier. It significantly reduced crystallite size and increased the specific surface area to 11.07 m^2^ g^−1^ (compared to 5.27 m^2^ g^−1^ for pure AMCT and 5.76 m^2^ g^−1^ for citric acid-processed samples), thereby enhancing the potential dissolution rate and bioavailability of the final product. Crucially, LC-MS and thermal analyses confirmed that malic acid serves strictly as a process aid and is not incorporated into the final crystal lattice.

In conclusion, this work demonstrates that the synergy of green chemistry and AI-driven optimization offers a robust, cost-effective, and GMP-compliant alternative to traditional AMCT manufacturing. The developed framework is readily adaptable to other pharmaceutical crystallization systems, paving the way for smarter and more sustainable drug production.

## Author contributions

Mustafa Fatih Ergin: conceptualization, methodology, software, validation, formal analysis, investigation, resources, data curation, writing – original draft, writing – review & editing, visualization, supervision, project administration, funding acquisition. Hasniye Yaşa: conceptualization, methodology, validation, formal analysis, investigation, writing – original draft, writing – review & editing. Hülya Çelik Onar: conceptualization, methodology, validation, investigation, resources, writing – original draft, writing – review & editing, supervision. All authors have read and agreed to the published version of the manuscript.

## Conflicts of interest

There are no conflicts to declare.

## Supplementary Material

RA-016-D5RA09898J-s001

## Data Availability

The data supporting this article have been included as part of the supplementary information (SI). Supplementary information: additional experimental details, ANN model parameters, Taguchi design matrices, extended characterization results (XRD, FTIR, DSC, BET, LC-MS), and raw optimization data. See DOI: https://doi.org/10.1039/d5ra09898j.
