# Supplementary material for: Green manufacturing of amoxicillin trihydrate: a malic acid-assisted crystallization framework enhanced by Taguchi–ANN optimization
Source: RSC Adv. 2026 Mar 17;16(16):14793–805. doi: 10.1039/d5ra09898j (PMC12994719; doi:10.1039/d5ra09898j)
Supplement: RA-016-D5RA09898J-s001 [file RA-016-D5RA09898J-s001.pdf]

Supplementary Information (SI) for RSC Advances.
This journal is © The Royal Society of Chemistry 2026

Supporting Information (SI)

Green Manufacturing of Amoxicillin Trihydrate: A Malic Acid-Assisted Crystallization Framework Enhanced by Taguchi–ANN Optimization

M.Fatih ERGİN[a*],Hasniye YAŞA[b*], Hülya ÇELİK ONAR[b]
Corresponding Author: mfergin@iuc.edu.tr

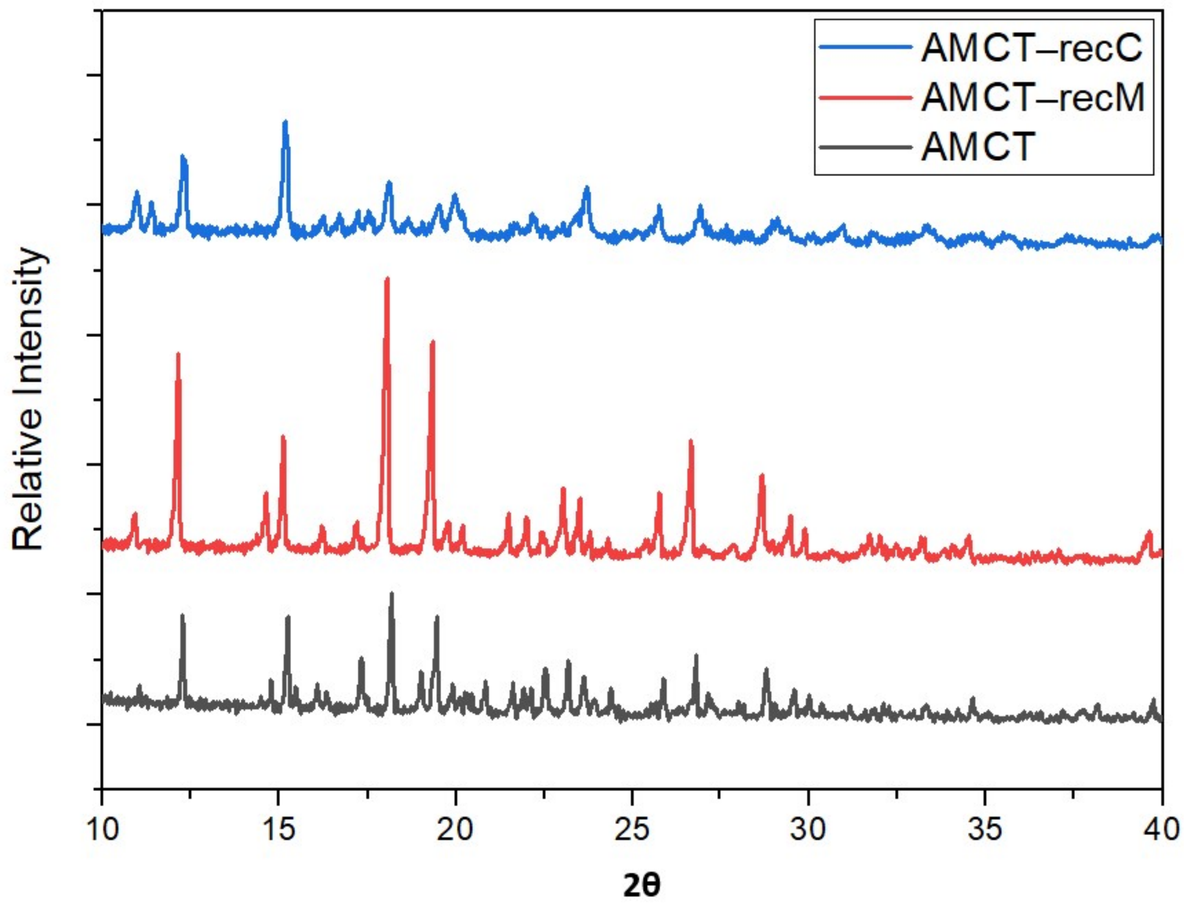

Figure S1. XRD Comparison of AMCT–recM and AMCT–recC

X-ray diffraction patterns comparing AMCT recrystallized with malic acid (AMCT–recM) and citric acid (AMCT–recC). The patterns reveal peak shifts and crystallinity changes in AMCT–recM, whereas AMCT–recC closely resembles pure AMCT, indicating that citric acid had minimal structural influence on the AMCT crystal lattice, whereas malic acid induced clear peak shifts and enhanced crystallinity.

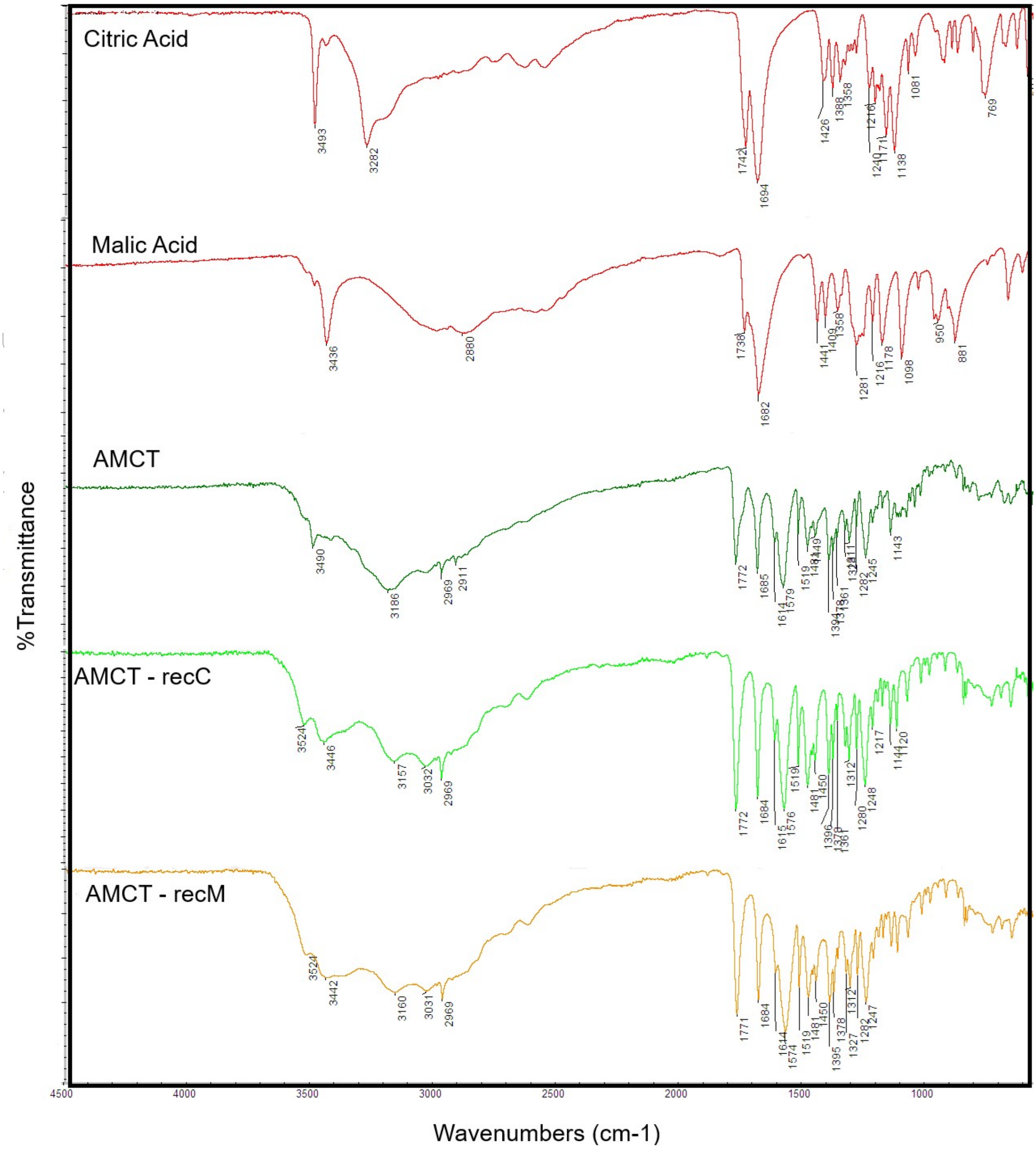

Figure S2. FTIR Spectra of AMCT, AMCT–recM, AMCT–recC, and Pure Acids

The FTIR spectra demonstrate the preservation of characteristic functional groups of AMCT in both recrystallized samples. No new peaks or significant shifts were detected, particularly in the carbonyl (1700–1600 cm^{-1}) and hydroxyl regions (3200–3400 cm^{-1}), confirming the absence of co-crystallization or salt formation.

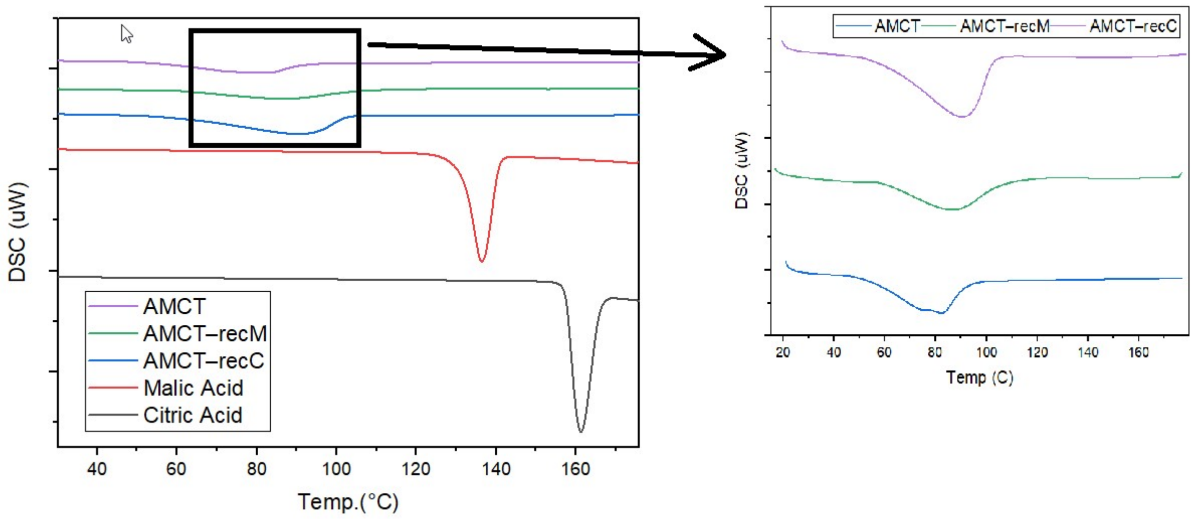

Figure S3. DSC Thermograms of AMCT–recM, AMCT–recC, and Pure Acids

DSC thermograms show that the dehydration and melting transitions of AMCT remain unaltered after recrystallization with either acid. The absence of the acid-specific thermal events confirms no incorporation of malic or citric acid into the crystal structure.

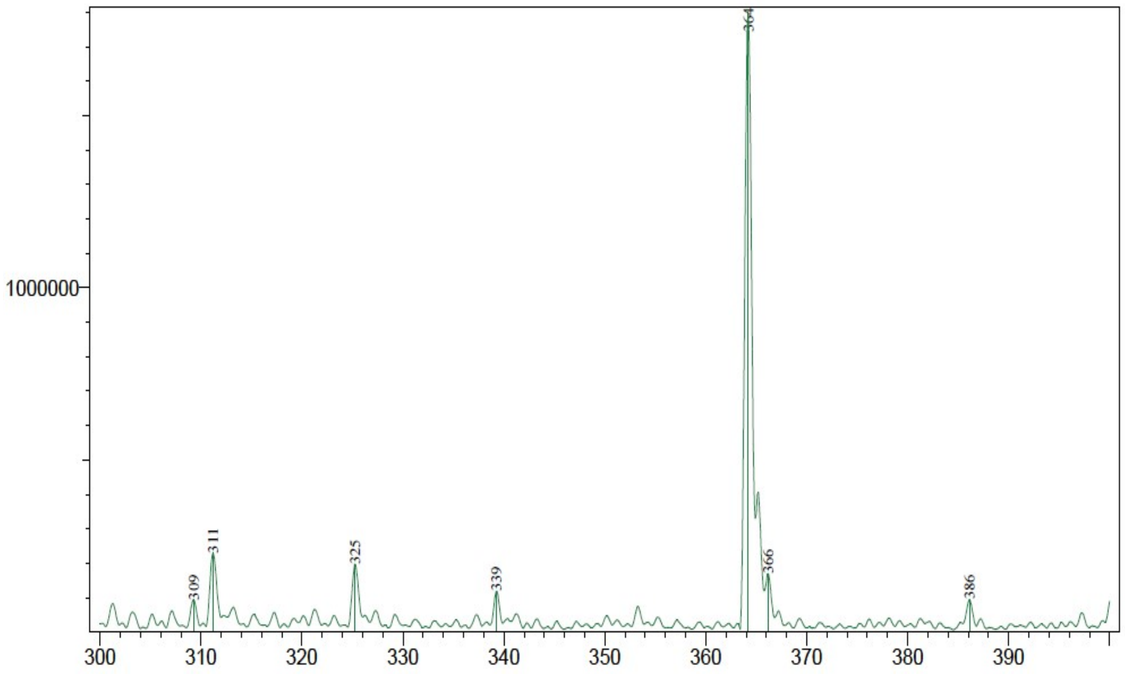

Figure S4. LC-MS Spectra of AMCT–recC

The LC-MS spectra of AMCT–recC revealed a dominant peak at m/z 364, corresponding to protonated AMCT. No secondary peaks attributable to citric acid, its fragments, or degradation products were detected, confirming chemical purity and the absence of molecular interactions.

Experimental Data for Taguchi and ANN Modeling

The supplementary dataset provided includes all experimental results used for both Taguchi optimization and artificial neural network (ANN) modeling. Specifically:

The Taguchi dataset comprises 18 experiments based on an L18 orthogonal array, used to identify the most influential parameters affecting the crystallization of amoxicillin trihydrate (AMCT).

The ANN dataset extends the experimental matrix to 54 data points, including the original 18 Taguchi trials and 36 additional independent batch crystallization experiments. These supplementary data points were experimentally obtained within the same design space to improve model generalizability and provide high-resolution data for robust training.

All 54 experiments were conducted under controlled and reproducible conditions. This enriched experimental dataset forms the foundation for the development, training, and validation of the ANN model, allowing accurate prediction of crystallization yield and purity across a continuous parameter space.

This dual-layered data approach enables hybrid optimization by combining the statistical efficiency of the Taguchi method with the nonlinear predictive capabilities of ANN modeling, supporting both experimental insight and computational process intensification.

Table S1. Taguchi L18 orthogonal array and experimental response data used for statistical screening..

pH	Mixing	Time	Cons	Efficiency (%)	Purity (%)
5	500	30	1.5	33.71	97.63
5	500	60	2	56.46	98.73
5	500	120	2.5	51.06	98.58
5	1000	30	1.5	36.16	97.82
5	1000	60	2	47.32	98.78
5	1000	120	2.5	45.13	99.43
5	1500	30	2	31.81	98.97
5	1500	60	2.5	50.4	99.38
5	1500	120	1.5	35.76	98.58
5.5	500	30	2.5	53.82	98.76
5.5	500	60	1.5	50.28	98.8
5.5	500	120	2	51.44	98.76
5.5	1000	30	2	41.11	98.9
5.5	1000	60	2.5	61.82	99.18
5.5	1000	120	1.5	41.46	98.79
5.5	1500	30	2.5	44.04	99.74
5.5	1500	60	1.5	39.71	98.88
5.5	1500	120	2	40.45	99.37

Table S2. Extended experimental dataset (54 points) used for training, validating, and testing the ANN model.

pH	Mixing	Time	Cons	Efficiency (%)	Purity (%)
5	500	30	1.5	33.71%	97.63%
5	500	60	1.5	47.04%	98.42%
5	500	120	1.5	48.81%	98.64%
5	500	30	2	39.38%	97.86%
5	500	60	2	56.46%	98.73%
5	500	120	2	54.49%	98.85%
5	500	30	2.5	52.26%	98.53%
5	500	60	2.5	60.27%	98.92%
5	500	120	2.5	51.06%	98.58%
5	1000	30	1.5	36.16%	97.82%
5	1000	60	1.5	42.33%	98.47%
5	1000	120	1.5	39.64%	98.56%
5	1000	30	2	34.09%	98.41%
5	1000	60	2	47.32%	98.78%
5	1000	120	2	42.68%	98.68%
5	1000	30	2.5	50.63%	98.69%
5	1000	60	2.5	58.98%	99.15%
5	1000	120	2.5	45.13%	99.43%
5	1500	30	1.5	32.26%	98.20%
5	1500	60	1.5	37.35%	98.62%
5	1500	120	1.5	35.76%	98.58%
5	1500	30	2	31.81%	98.97%
5	1500	60	2	39.76%	99.17%
5	1500	120	2	38.41%	99.38%
5	1500	30	2.5	38.16%	99.44%
5	1500	60	2.5	50.40%	99.38%
5	1500	120	2.5	42.38%	99.38%
5.5	500	30	1.5	44.45%	98.51%
5.5	500	60	1.5	50.28%	98.80%
5.5	500	120	1.5	40.46%	98.27%
5.5	500	30	2	48.75%	98.69%
5.5	500	60	2	54.83%	98.99%
5.5	500	120	2	51.44%	98.76%
5.5	500	30	2.5	53.82%	98.76%
5.5	500	60	2.5	57.62%	99.06%
5.5	500	120	2.5	55.65%	98.98%
5.5	1000	30	1.5	39.37%	98.38%
5.5	1000	60	1.5	45.21%	98.64%
5.5	1000	120	1.5	41.46%	98.79%
5.5	1000	30	2	41.11%	98.90%
5.5	1000	60	2	54.53%	98.83%

5.5	1000	120	2	44.36%	98.93%
5.5	1000	30	2.5	53.78%	99.07%
5.5	1000	60	2.5	61.82%	99.18%
5.5	1000	120	2.5	46.83%	99.32%
5.5	1500	30	1.5	35.46%	98.49%
5.5	1500	60	1.5	39.71%	98.88%
5.5	1500	120	1.5	37.32%	99.13%
5.5	1500	30	2	35.41%	99.14%
5.5	1500	60	2	45.23%	99.58%
5.5	1500	120	2	40.45%	99.37%
5.5	1500	30	2.5	44.04%	99.74%
5.5	1500	60	2.5	55.76%	99.87%
5.5	1500	120	2.5	42.82%	99.74%

Table S3. Detailed comparison of XRD peak parameters for pure AMCT and malic acid-assisted AMCT (AMCT–recM).

2θ (°)	d-spacing	Pure AMCT I/I₀	AMCT–recM I/I₀	Pure AMCT FWHM (°)	AMCT–recM FWHM (°)	Pure AMCT Size (nm)	AMCT–recM Size (nm)	Interpretation
10.91	8.1063	-	119.23	-	0.1176	-	70.1	Smaller crystallites formed with AMCT–recM
12.13	7.2879	286.01	685.61	0.07	0.1045	118.02	82.6	Improved crystallinity
14.62	6.0544	-	181.56	-	0.1174	-	73.5	Enhanced structural organization
15.11	5.8596	-	401.46	-	0.0997	-	87.2	Enhanced structural uniformity
16.22	5.4613	-	93.15	-	0.1066	-	81.5	Moderate crystallinity
17.18	5.1578	-	89.96	-	0.1534	-	60.1	Broad peak indicating smaller crystals
18.03	4.9161	379.28	1000	0.0972	0.1107	82.5	74.3	Highest intensity peak
19.31	4.5935	-	738.91	-	0.1019	-	80.1	Lattice distortion evident
19.75	4.4907	-	108.29	-	0.1446	-	67.9	Small and less ordered crystals
20.17	4.3983	-	96.21	-	0.1049	-	78.3	Improved structure
21.47	4.1353	-	139.88	-	0.0931	-	85.1	Uniform crystal formation
22.44	3.9586	-	77.41	-	0.1466	-	67.5	Reduced crystallinity
23.02	3.8606	-	234.13	-	0.1264	-	72.3	Moderate uniformity
23.78	3.7388	-	85.64	-	0.0709	-	102.1	Enhanced lattice structure
25.36	3.5086	-	47.83	-	0.2561	-	32.6	Formation of smaller, less ordered crystals
26.66	3.341	-	397.46	-	0.1252	-	74.2	Strong crystallinity
27.03	3.2957	-	35.87	-	0.2155	-	43.1	Minor crystal phase
28.67	3.1116	-	293.76	-	0.1492	-	69.4	High uniformity
34.5	2.5979	-	80.05	-	0.1354	-	71.1	Improved lattice organization
39.58	2.275	-	106.71	-	0.2136	-	50.2	Smaller crystals
